# Phase‐Specific Contributions and Interactions of the Left and Right Posterior Middle Temporal Gyri in Vocal Feedback Control: Evidence From Dual‐Site TMS

**DOI:** 10.1002/hbm.70390

**Published:** 2025-10-29

**Authors:** Qingqing Liu, Jiating Li, Shuzhi Zhao, Mingyun Chen, Xin Huang, Dongxu Liu, Jingting Li, Xiuqin Wu, Yongxue Li, Xi Chen, Peng Liu, Guangyan Dai, Hanjun Liu

**Affiliations:** ^1^ Department of Rehabilitation Medicine, The First Affiliated Hospital Sun Yat‐sen University Guangzhou China; ^2^ CAS Key Laboratory of Human‐Machine Intelligence‐Synergy Systems, Shenzhen Institute of Advanced Technology Chinese Academy of Sciences Shenzhen China; ^3^ Guangdong Provincial Key Laboratory of Brain Function and Disease, Zhongshan School of Medicine Sun Yat‐sen University Guangzhou China; ^4^ Guangdong Provincial Clinical Research Center for Rehabilitation Medicine Guangzhou China; ^5^ Guangdong Engineering and Technology Research Centre of Rehabilitation Medicine and Translation Guangzhou China

**Keywords:** auditory feedback, continuous theta burst stimulation, interhemispheric interaction, posterior middle temporal gyrus, speech motor control

## Abstract

The posterior middle temporal gyrus (pMTG) has been implicated in sensorimotor control of speech production, but the causality underlying this relationship remains largely unclear. The present event‐related potential study employed dual‐site continuous theta burst stimulation (c‐TBS) over the left and right pMTGs concurrently to investigate their causal roles and interhemispheric interactions in vocal feedback control. Following bilateral c‐TBS, unilateral c‐TBS paired with contralateral sham stimulation, or bilateral sham stimulation over the left and right pMTGs, 24 healthy young adults produced sustained vocalizations while exposed to unexpected pitch perturbations (±200 cents) in auditory feedback. Compared to sham stimulation, c‐TBS over the left, right, or bilateral pMTG significantly reduced the magnitudes and shortened the latencies of vocal compensations, paralleled by enhanced P2 responses that received contributions from distinct fronto‐tempo‐parietal networks. In contrast, reduced N1 responses were observed only following bilateral pMTG stimulation. Our findings not only provide the first causal evidence for bilateral pMTG involvement in vocal feedback control but also reveal a phase‐specific interhemispheric interaction, transitioning from bilateral coordination during early error detection to unilateral sufficiency during later motor correction. These insights pave new avenues for developing novel multi‐site neuromodulation protocols to optimize speech rehabilitation.

## Graphical abstract

This study demonstrates the causal roles of the left and right posterior middle temporal gyri in vocal feedback control and reveals a phase‐specific interhemispheric mechanism that transitions from coordinated error detection to independent correction, offering novel neuromodulation strategies for speech rehabilitation.

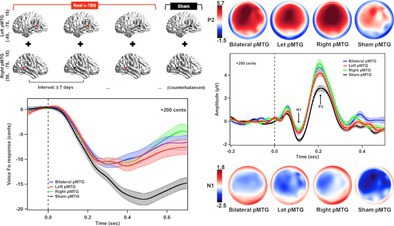

## 1 Introduction

Accurate speech production relies on the ability to continuously monitor and adjust vocal output, a process that integrates sensory feedback, particularly auditory feedback, with motor commands. Specifically, the brain detects discrepancies between intended and actual speech sounds and generates corrective adjustments, as evidenced by compensatory vocal responses to perturbations of fundamental frequency, intensity, or formant frequency in auditory feedback (Bauer et al. 2006; Burnett et al. 1998; Houde and Jordan 1998). Neuroimaging studies have identified a distributed network that supports this feedback‐based control of vocal production, including the dorsolateral prefrontal cortex (DLPFC), premotor cortex (PMC), primary motor cortex (M1), supplementary motor area (SMA), inferior frontal gyrus (IFG), superior temporal gyrus (STG), inferior parietal lobule (IPL)/supramarginal gyrus (SMG), cerebellum, and basal ganglia (Behroozmand, Ibrahim, et al. 2015; Behroozmand, Shebek, et al. 2015; Chang et al. 2013; Kort et al. 2016; Liu et al. 2023, 2020; Parkinson et al. 2012; Tourville et al. 2008; Zarate and Zatorre 2008).

While the STG is traditionally considered the principal region for processing voice auditory feedback errors (Guenther et al. 2006; Liu et al. 2023; Parkinson et al. 2012; Tourville and Guenther 2011), emerging evidence indicates that the posterior middle temporal gyrus (pMTG) also plays a role in this control process. The pMTG, located dorsally along the superior temporal sulcus and ventrally adjacent to the inferior temporal sulcus (Briggs et al. 2021), has been traditionally associated with semantic processing (Hickok and Poeppel 2007; Jackson 2021). However, functional neuroimaging studies have shown activation of the pMTG in response to perturbed auditory feedback during vocal production (Kort et al. 2016; Parkinson et al. 2012; Ranasinghe et al. 2019; Sares et al. 2020). Notably, the magnitude of vocal compensation was negatively correlated with high‐gamma power in the left pMTG in healthy adults (Kort et al. 2016) and positively correlated with high‐gamma power in the right pMTG in patients with Alzheimer's disease (AD) (Ranasinghe et al. 2019). Lesion evidence from stroke patients with aphasia further showed that the extent of damage to the left MTG was significantly correlated with diminished vocal compensation within 50–150 ms following pitch perturbations (Behroozmand et al. 2018). In addition, event‐related potentials (ERPs) studies have implicated the MTG in the generation of cortical responses to vocal pitch perturbations, with the left MTG contributing to the N1 response and the bilateral MTG involvement in the P2 response (Dai, Chen, et al. 2022; Dai, Wang, et al. 2022; Guo et al. 2017; Huang et al. 2016). These two components have been functionally linked to different stages of vocal feedback control, where the N1 response reflects early detection of feedback errors and the P2 response reflects subsequent corrective processes (Behroozmand et al. 2011; Guo et al. 2017; Hawco et al. 2009; Scheerer et al. 2013). These findings suggest that, beyond the STG, the pMTG may also be involved in auditory feedback control of speech production.

Nevertheless, converging neuroimaging evidence linking the pMTG to vocal feedback control remains largely correlational, leaving open the causality underlying this relationship. One promising approach to examining such causal relationships is continuous theta burst stimulation (c‐TBS), a patterned form of transcranial magnetic stimulation (TMS) that induces enduring inhibitory effects on cortical excitability (Huang et al. 2005). This approach builds on a growing body of TMS studies that have revealed causal contributions of various brain regions to vocal feedback control. For example, c‐TBS over the left DLPFC enhanced vocal compensations for pitch perturbations and reduced P2 responses (Liu et al. 2020). In contrast, c‐TBS over the left SMA and left/right SMG reduced both vocal compensations and P2 responses (Dai, Chen, et al. 2022; Dai, Wang, et al. 2022; Li, Chang, et al. 2023; Li, Zhu, et al. 2023), while c‐TBS over the right STG and cerebellum reduced vocal compensations but enhanced P2 responses (Lin et al. 2022; Liu et al. 2023). These findings highlight the regional specificity and complexity of cortical contributions to vocal feedback control. However, whether the pMTG exerts a causal influence on the neurobehavioral processing of voice feedback errors has yet to be determined.

Moreover, while previous studies have implicated the left pMTG (Behroozmand et al. 2018; Guo et al. 2017; Huang et al. 2016), right pMTG (Dai, Chen, et al. 2022; Dai, Wang, et al. 2022), or both (Kort et al. 2016; Parkinson et al. 2012) in vocal pitch regulation, the precise roles and potential interactions between the left and right pMTGs remain poorly understood. Evidence from other regions suggests that unilateral stimulation effects may not be confined to the targeted hemisphere but may reflect interhemispheric dynamics. For example, c‐TBS over the right STG, but not the left, reduced vocal compensations and enhanced P2 responses (Liu et al. 2023), while c‐TBS over either the left or right SMG produced similar reductions in vocal compensations and P2 responses (Li, Chang, et al. 2023; Li, Zhu, et al. 2023). Such findings suggest that unilateral stimulation may be shaped by interhemispheric interactions, such as inhibitory competition, compensatory recruitment, or functional independence. For example, interhemispheric inhibition occurs in motor and somatosensory cortices during unimanual movements and tactile tasks (Boddington and Reynolds 2017; Brodie et al. 2014; Denyer et al. 2024), while interhemispheric compensation allows contralateral regions to offset functional deficits following brain injury (Andoh and Martinot 2008; Hartwigsen et al. 2013; Li, Guo, et al. 2015; Li, Qu, et al. 2015; Salazar et al. 2018; Vuilleumier et al. 1996; Zhang et al. 2025). In addition, interhemispheric independence has been observed in the IFG and SMG during phonological tasks, where bilateral stimulation yields functional outcomes similar to unilateral stimulation (Hartwigsen, Baumgaertner, et al. 2010; Hartwigsen, Price, et al. 2010). These findings raise the possibility that the left and right pMTGs may contribute to vocal feedback control through interhemispheric interactions, with their contributions varying depending on whether coordination, compensation, or independence is required.

To address these gaps, the present study employed dual‐site c‐TBS to investigate the causal contributions and interhemispheric interactions between the left and right pMTGs in vocal feedback control. By concurrently stimulating both hemispheres, this approach allowed us to determine whether the pMTG operates unilaterally or bilaterally in the processing of vocal feedback errors and to characterize the nature of interhemispheric interactions. Previous studies have validated dual‐site concurrent TMS as a reliable method for probing interhemispheric interactions (Dambeck et al. 2006; Hartwigsen, Baumgaertner, et al. 2010; Hartwigsen, Price, et al. 2010). The frequency‐altered feedback (FAF) paradigm (Burnett et al. 1998) was used to assess the neurobehavioral effects of c‐TBS over the pMTG on vocal feedback control, where participants produced sustained vowels while hearing unexpected pitch perturbations in their auditory feedback. Vocal responses and cortical ERPs, particularly the N1 and P2 components, were measured and compared across the conditions. These measures have been previously used to examine the causal effects of c‐TBS on auditory‐vocal integration (Dai, Chen, et al. 2022; Dai, Wang, et al. 2022; Li, Chang, et al. 2023; Li, Zhu, et al. 2023; Liu et al. 2023, 2020). In addition, source localization analysis was performed to identify the neural generators associated with changes in N1 and/or P2 responses between active and sham stimulations (Liu et al. 2023, 2020). Within this design, we hypothesized two primary patterns reflecting different modes of interhemispheric interaction. If significant effects were observed only following unilateral stimulation over the left or right pMTG, this would suggest that either pMTG is sufficient to support auditory‐vocal integration, consistent with functional independence or redundancy. If significant effects were observed only following bilateral pMTG stimulation, this would suggest that coordinated bilateral engagement is required for auditory‐vocal integration, reflecting interhemispheric coordination.

## 2 Methods

### 2.1 Subjects

Twenty‐four right‐handed, native‐Mandarin speaking college students from Sun Yat‐sen University of China (13 female and 11 male; age: 22.54 ± 2.02 years) participated in the present study. Exclusion criteria included pregnancy, use of neuropsychiatric medications, prior musical training, presence of implanted medical devices, or a history of speech, language, hearing, or neurological disorders. In addition, participants had no contraindications to TMS or magnetic resonance imaging (MRI). All participants provided written informed consent and received monetary compensation for their participation. Data from two participants were excluded because their data quality did not meet the criteria for inclusion (see below), resulting in a final sample of 22 participants (13 female and 9 male, aged 22.36 ± 1.97 years). The study protocol was approved by the Institutional Review Board of The First Affiliated Hospital of Sun Yat‐sen University and adhered to the Code of Ethics of the World Medical Association.

### 2.2 Structural MRI Data Acquisition

High‐resolution structural images were acquired using a 3T MRI (Siemens, Germany) scanner prior to the TMS administration to enable precise localization of stimulation targets. T1‐weighted images were obtained using a magnetization‐prepared rapid gradient‐echo (MPRAGE) sequence with the following parameters: repetition time = 2300 ms, echo time = 2.19 ms, slice thickness = 1 mm, field of view = 256 × 256 mm^2^, flip angle = 9^o^.

### 2.3 Transcranial Magnetic Stimulation

Magnetic stimulation was administered using a MagTD TMS instrument (YIRUIDE Co., Wuhan, China) with a 7‐cm outer diameter figure‐of‐eight coil. The stimulation intensity was set at 80% of the active motor threshold (AMT), defined as the minimum stimulus intensity required to induce motor evoked potentials (MEPs) greater than 200 μv from the right first dorsal interosseous muscle in at least 5 out of 10 consecutive trials during single‐pulse TMS over the M1 at about 10% voluntary contraction. Target sites for stimulation were identified using neuronavigation software (Visor 2.0, ANT Neuro, Netherlands) combined with a Polaris Spectra motion tracking system (NDI, Canada). High‐resolution structural MRI data from each participant were loaded into the software and normalized to Montreal Neurological Institute (MNI) space. Stimulation sites at the left pMTG (*x* = −55, *y* = −75, *z* = 15) and right pMTG (*x* = 55, *y* = −75, *z* = 15) were identified using group coordinates from a previous study (Kort et al. 2016). These coordinates were slightly adjusted for each participant based on individual anatomical markers to ensure accurate and precise stimulation. A standard c‐TBS protocol was employed, consisting of trains of 3‐pulse bursts delivered at 50 Hz with bursts being repeated every 200 ms for a total of 600 pulses (Huang et al. 2005). Active stimulation was delivered by placing the coil tangential to the skull surface, while sham stimulation was delivered by placing the coil at 90 ***°*** to the skull surface.

### 2.4 Experimental Design

**FIGURE 1:**
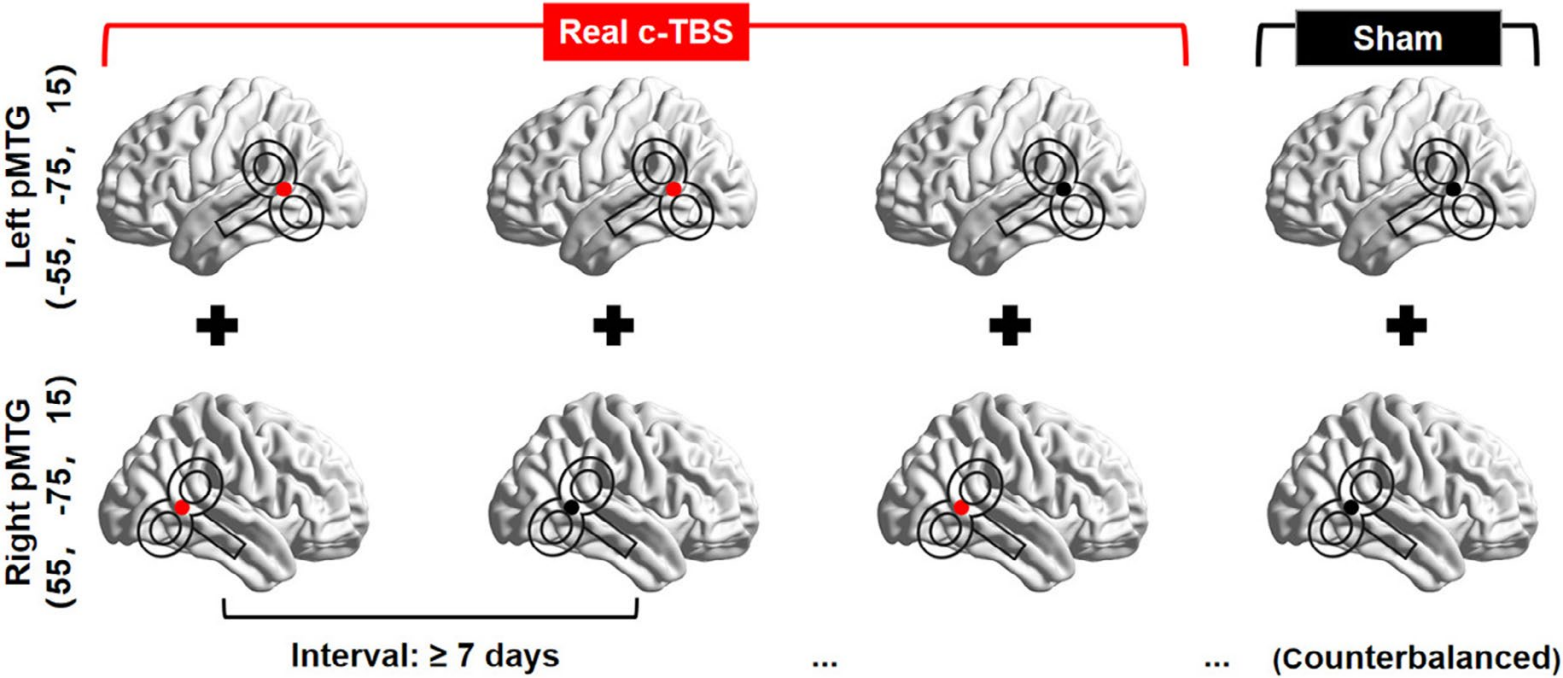
Participants underwent one of four stimulation sessions (from left to right): (1) dual‐site real c‐TBS over the bilateral pMTG, (2) real c‐TBS over the left pMTG coupled with sham c‐TBS over the right SMG, (3) real c‐TBS over the right SMG coupled with sham c‐TBS over the left SMG, and (4) dual‐site sham c‐TBS over the bilateral pMTG. The TMS coil with a red dot represents real stimulation, while the TMS coil with a black dot represents sham stimulation. During sham stimulation, the TMS coil was oriented perpendicular to the scalp, with only the coil edge touching the scalp. The session order was counterbalanced across participants, with each session conducted on separate days at least 1 week apart.

In this randomized, cross‐sectional study, participants underwent four stimulation sessions that applied dual‐site c‐TBS over the left and right pMTG concurrently (see Figure 1): (1) active c‐TBS over both the left and right pMTGs (bilateral pMTG), (2) active c‐TBS over the left pMTG and sham c‐TBS over the right pMTG (left pMTG), (3) sham c‐TBS over the left pMTG and active c‐TBS over the right pMTG (right pMTG), and (4) sham c‐TBS over both the left and right pMTGs (sham pMTG). The order of these four sessions was counterbalanced across all participants, with each session conducted on separate days at least 1 week apart to prevent carryover effects. The mean percentage of maximum stimulator output (MSO ± SD) across participants was 30.00% ± 7.98% MSO for bilateral pMTG, 28.34% ± 7.87% MSO for left pMTG, 29.55% ± 8.33% MSO for right pMTG, and 29.64% ± 8.20% MSO for sham pMTG.

Immediately following each stimulation session, all participants performed the FAF‐based vocal production task in a sound‐treated booth. They were instructed to vocalize the vowel sound/u/for 3–4 s at their habitual pitch and loudness levels upon the appearance of a black cross on the computer monitor and maintained their vocalizations steady until the black cross disappeared. During each vocalization, the participant's voice was pseudo‐randomly pitch‐shifted by either +200 cents or −200 cents (100 cents = 1 semitone) for 200 ms. Each vocalization consisted of two pitch perturbations, with the first occurring 1200–1500 ms after vocalization onset, followed by a second perturbation with an inter‐stimulus interval of 1200–1500 ms. The direction of each perturbation (either upward or downward) was independently pseudo‐randomized across the entire session, such that the two perturbations within a single vocalization could occur in either the same or opposite directions. All participants were required to take a break of 2–3 s between each vocalization to prevent vocal fatigue. Each participant produced 100 consecutive vocalizations, including a total of 200 trials that included 100 trials for −200 cents perturbations and 100 trials for +200 cents perturbations in an unpredictable sequence. The experimental parameters of the vocal production task were identical across all stimulation sessions.

### 2.5 Data Recording

Voice signals were recorded using a dynamic microphone (DM2200, Takstar Inc.) and transmitted to an Eventide Eclipse Harmonizer via a MOTU Ultralite Mk3 Firewire audio interface. A Max/MSP software program (v.6.0 by Cycling 74) was custom‐developed to control the Harmonizer for pitch‐shifting the voice signals, to generate transistor‐transistor logic (TTL) control pulses to mark the perturbation onset, and to provide visual cues to guide the participants in starting and ending their vocalizations. Finally, the pitch‐shifted voices were amplified by an ICON NeoAmp headphone amplifier and delivered to participants through inserted earphones (ER‐1, Etymotic Research Inc.). Both the original and pitch‐shifted voice signals, as well as the TTL control pulses, were digitized at a sampling rate of 10 kHz by a PowerLab A/D converter (ML880, AD Instruments) and recorded using LabChart software (v.7.0 by AD Instruments).

Electroencephalography (EEG) data were simultaneously scalp‐recorded during the vocal production task using a 64‐electrode Geodesic Sensor Net connected to a Net Amps 400 amplifier (Electrical Geodesics Inc., Eugene, OR). Impedance levels of all individual sensors were adjusted to less than 50 kΩ throughout the online recording (Ferree et al. 2001). TTL control pulses were transmitted via a DIN synch cable to synchronize the onset of the pitch perturbation with the EEG data for subsequent ERP analysis. EEG signals were referenced to the vertex (Cz) during online recording (Ferree et al. 2001) and recorded at a sampling rate of 1 kHz using NetStation software (v. 5.4.2, Electrical Geodesics Inc., Eugene, OR).

### 2.6 Data Analysis

As previously described (Liu et al. 2020, 2010), vocal responses to pitch perturbations were analyzed using a custom‐developed software program (IGOR PRO, v.6.0, Wavemetrics Inc.). In brief, the voice *f*_o_ contour in Hertz was first extracted using Praat software (Boersma 2001) and converted to a cent scale using the following formula: cents = 100 × (12 × log2(*f*_o_/reference)) (reference = 195.997 Hz [G3 note]). Next, the voice *f*_o_ contour in cents was segmented into epochs ranging from −100 to +700 ms with respect to the onset of pitch perturbations. All segmented trials were inspected to reject bad trials corrupted by vocal interruptions or signal processing errors. Finally, artifact‐free trials were averaged and baseline‐corrected (−100 to 0 ms) to generate an overall vocal response. The magnitude and latency of vocal responses were defined as the peak amplitude in cents and the peak time in ms when the voice *f*_o_ contours reached their maximum or minimum values, respectively.

EEG data were offline analyzed using NetStation software. First, they were band‐pass filtered between 1 and 20 Hz to reduce low‐frequency drift and high‐frequency noise while preserving the spectral range of the ERP components of interest (Behroozmand, Ibrahim, et al. 2015; Luck 2014; Tanner et al. 2015) and segmented into epochs ranging from −200 to +500 ms relative to the onset of pitch perturbations. Artifact detection was then performed by rejecting trials where the amplitude exceeded ±55 μV in the moving average over an 80‐ms window or if more than 10 bad channels were affected by artifacts. Any files with more than 20% of epochs containing artifacts were discarded. Additional visual inspection was conducted to ensure that all individual trials were appropriately rejected. Finally, artifact‐free trials were re‐referenced to the average of electrodes on each mastoid, averaged, and baseline‐corrected (−200 to 0 ms) to generate an overall ERP response. Considering that ERP responses to pitch perturbations are typically prominent in the front and central regions (Guo et al. 2016; Scheerer et al. 2013), 24 electrodes were selected from three regions of interest (ROIs) for statistical analyses (Dai, Chen, et al. 2022; Dai, Wang, et al. 2022; Liu et al. 2020): frontal area (AF3, AFz, AF4, F5, F3, F1, Fz, F2, F4, F6), fronto‐central area (FC5, FC3, FC1, FCz, FC2, FC4, FC6), central area (C5, C3, C1, Cz, C2, C4, C6). The amplitudes and latencies of the N1 and P2 components were measured from the averaged ERPs within each ROI, with the N1 defined as the negative peak amplitude within the time window of 80–180 ms and the P2 defined as the positive peak amplitude within the time window of 160–280 ms after perturbation onset.

### 2.7 Source Localization

Source localization was performed using Brainstorm software (v. 3.2) (Tadel et al. 2011) to characterize changes in the neural networks underlying vocal pitch regulation following c‐TBS over the pMTG. For each participant, individual head models were constructed by co‐registering ERP electrode positions with each participant's structural MRI using a mutual information algorithm (Maes et al. 1997). The forward model was computed using the boundary element method (Haueisen et al. 1997) to generate a lead‐field matrix necessary for localizing EEG sources. To solve the inverse problem, we applied a linear, distributed source model using minimum norm estimation (Hamalainen and Ilmoniemi 1994) to estimate the spatial distribution of cortical activity associated with the ERP responses. Source activity was constrained to the cortical surface and anatomically parcellated using the Destrieux atlas to identify specific brain regions. Neural responses were analyzed within two time windows corresponding to the ERP components of interest: 80–180 ms for the N1 component and 160–280 ms for the P2 component. To evaluate the effects of c‐TBS, paired two‐sample t‐tests were conducted to compare neural activity between active and sham stimulations. Statistical significance was determined using a voxel‐wise threshold of *p* < 0.05, with corrections for multiple comparisons using the false discovery rate method.

### 2.8 Statistical Analysis

The normality of vocal and ERP responses was verified using the Kolmogorov–Smirnov test. They were analyzed using repeated‐measures analysis of variances (RM‐ANOVAs) in SPSS (v.20.0). The magnitudes and latencies of vocal responses were subjected to two‐way RM‐ANOVAs, including within‐subject factors of stimulation session (bilateral pMTG, left pMTG, right pMTG, and sham pMTG) and perturbation direction (−200 vs. +200 cents). The amplitudes and latencies of the N1 and P2 responses were subjected to three‐way RM‐ANOVAs, including within‐subject factors of stimulation session, perturbation direction, and electrode site (frontal, fronto‐central, and central). Any significant higher‐order interactions led to subsidiary RM‐ANOVAs analysis. Post hoc Bonferroni comparisons were conducted to correct multiple comparisons. When the sphericity assumption was violated, Greenhouse–Geisser correction was applied to adjust *p*‐values for multiple degrees of freedom. *p* < 0.05 was considered statistically significant, and effective sizes were reported as partial *η* ^2^ ($\eta_{p}^{2}$) for RM‐ANOVA effects and Cohen's d for pairwise comparison.

## 3 Results

### 3.1 Behavioral Findings

**FIGURE 2:**
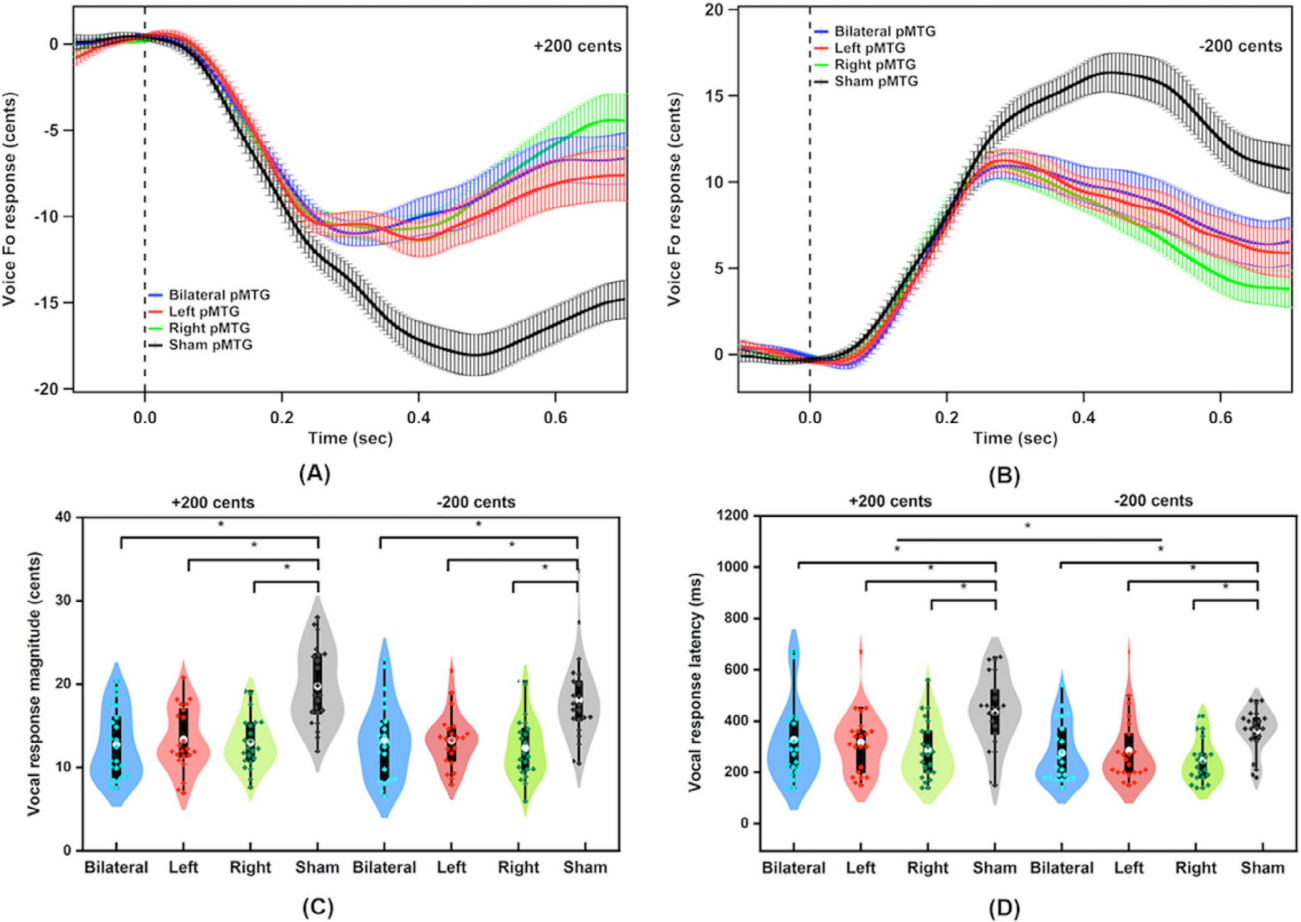
(A, B) Grand‐averaged voice *f*_o_ responses to pitch perturbations of +200 and −200 cents following c‐TBS over the bilateral pMTG (blue), left pMTG (red), right pMTG (green), and sham stimulation (black). Vertical bars across the voice *f*_o_ contours represent the standard errors of the mean vocal responses. (C, D) Violin plots of the peak magnitudes and times of vocal responses to pitch perturbations of +200 and −200 cents following c‐TBS over the bilateral pMTG (blue), left pMTG (red), right pMTG (green), and sham stimulation (black). The white dots and box plots represent the medians and ranges from the first to third quartiles of the data sets. The blue, red, green, and black dots represent the individual ERP responses to pitch perturbations. The asterisks indicate significant differences across the conditions.

Figure 2 shows the grand‐averaged voice *f*_o_ responses to pitch perturbations (A, B) and violin plots representing the statistical distributions of vocal responses (C, D) across conditions. A two‐way RM‐ANOVA conducted on the peak magnitudes of vocal responses showed a significant main effect of stimulation session (*F*(3, 63) = 39.248, *p* < 0.001, $\eta_{p}^{2}$ = 0.651). Post hoc analysis showed that sham pMTG resulted in larger vocal responses than bilateral pMTG (*p* < 0.001, Cohen's *d* = 1.433), left MTG (*p* < 0.001, Cohen's *d* = 1.366), and right pMTG (*p* < 0.001, Cohen's *d* = 1.513), respectively. However, no significant main effect was found for perturbation direction (*F*(1, 21) = 0.893, *p* = 0.335, $\eta_{p}^{2}$ = 0.041), nor was there a significant interaction between stimulation session and perturbation (*F*(3, 63) = 0.958, *p* = 0.418, $\eta_{p}^{2}$ = 0.044).

Regarding the peak times of vocal responses, there was a significant main effect of stimulation session (*F*(3, 63) = 9.356, *p* < 0.001, $\eta_{p}^{2}$ = 0.308), showing longer peak times of vocal responses following sham MTG compared to bilateral pMTG (*p* = 0.002, Cohen's *d* = 0.791), left pMTG (*p* = 0.012, Cohen's *d* = 0.792), and right pMTG (*p* < 0.001, Cohen's *d* = 1.069). A significant main effect of perturbation direction was also found (*F*(1, 21) = 11.334, *p* = 0.003, $\eta_{p}^{2}$ = 0.351), showing shorter peak times of vocal responses to downward perturbations than to upward perturbations. In contrast, the interaction between stimulation session and perturbation direction was not significant (*F*(3, 63) = 0.376, *p* = 0.771, $\eta_{p}^{2}$ = 0.018).

### 3.2 ERP Findings

**FIGURE 3:**
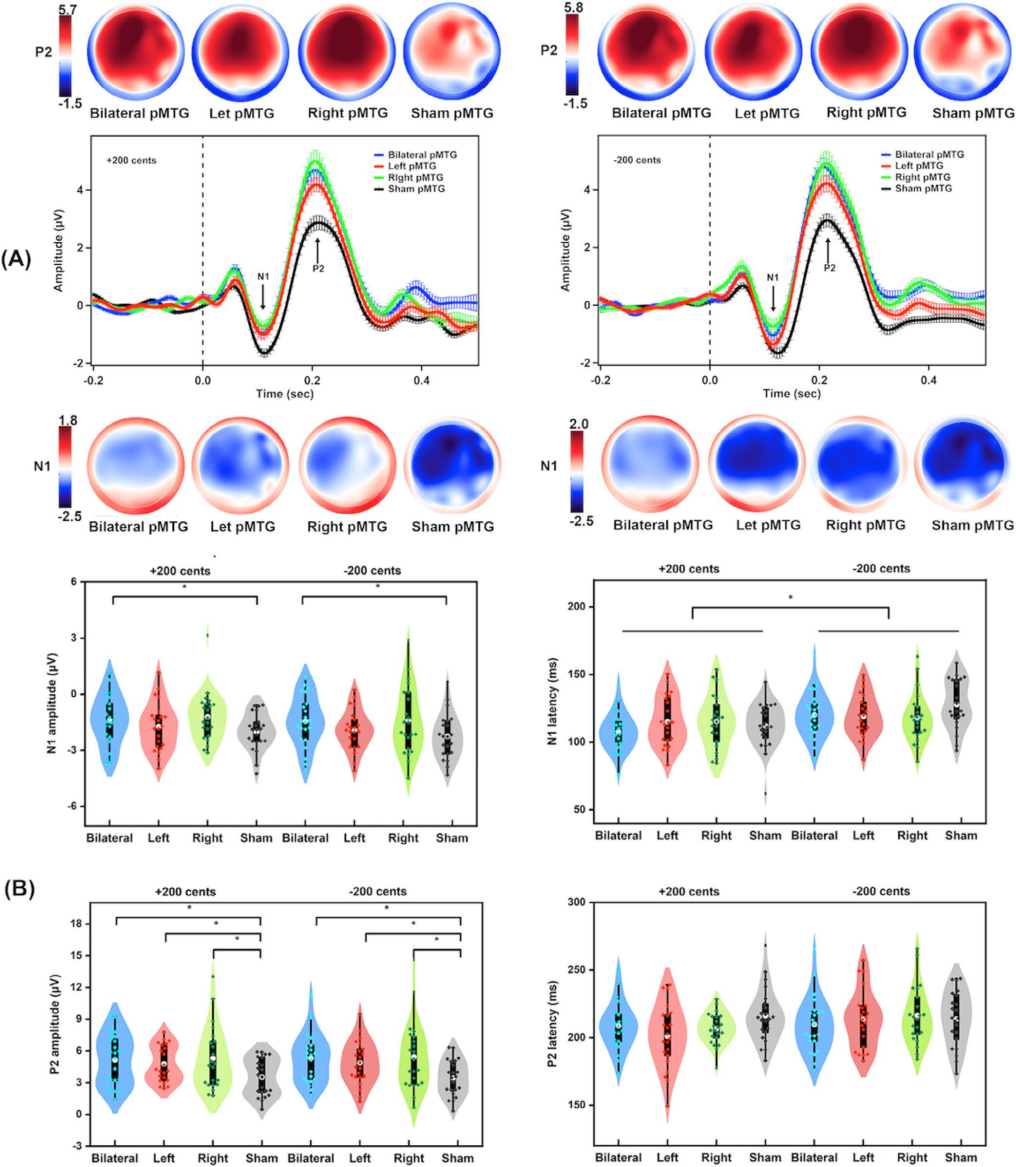
(A) Grand‐averaged ERP waveforms across 24 electrodes (middle) and topographical distribution maps of the N1 and P2 responses to pitch perturbations of +200 (left pannel) and −200 cents (right panel) following c‐TBS over the bilateral pMTG (blue), left pMTG (red), right pMTG (green), and sham stimulation (black). Vertical bars across the ERP waveforms indicate the standard errors of the mean ERP responses. (B) Violin plots of the amplitudes and latencies of the N1 and P2 responses to pitch perturbations of +200 and −200 cents following c‐TBS over the bilateral pMTG (blue), left pMTG (red), right pMTG (green), and sham stimulation (black). The white dots and box plots represent the medians and ranges from the first to third quartiles of the data sets. The blue, red, green, and black dots represent the individual ERP responses to pitch perturbations. The asterisks indicate significant differences across the conditions.

Figure 3 shows the grand‐averaged ERPs to pitch perturbations and topographical maps of N1 and P2 responses (A) and violin plots representing the statistical distributions of N1 and P2 responses (B) across conditions. A three‐way RM‐ANOVA conducted on the N1 amplitudes revealed a significant main effect of stimulation session (*F*(3, 63) = 3.270, *p* = 0.027, $\eta_{p}^{2}$ = 0.135), showing larger (more negative) N1 responses following sham pMTG compared to bilateral pMTG (*p* < 0.001, Cohen's *d* = 0.507). However, no significant main effects were found for electrode site (*F*(2, 42) = 3.389, *p* = 0.059, $\eta_{p}^{2}$ = 0.139) and perturbation direction (*F*(1, 21) = 1.337, *p* = 0.260, $\eta_{p}^{2}$ = 0.060), nor were there significant interactions among any three factors (*p*s > 0.3, all $\eta_{p}^{2}$ < 0.044).

Regarding the N1 latencies, there was a significant main effect of perturbation direction (*F*(1, 21) = 39.025, *p* < 0.001, $\eta_{p}^{2}$ = 0.650), indicating shorter peak times of N1 responses to upward perturbations than to downward perturbations. However, no significant main effect was found for stimulation session (*F*(3, 63) = 1.474, *p* = 0.230, $\eta_{p}^{2}$ = 0.066) or electrode site (*F*(2, 42) = 0.015, *p* = 0.946, $\eta_{p}^{2}$ = 0.001), nor were there significant interactions between any of these three factors (*p*s > 0.1, all $\eta_{p}^{2}$ < 0.119).

A three‐way RM‐ANOVA conducted on the P2 amplitudes revealed a significant main effect of stimulation session (*F*(3, 63) = 10.533, *p* < 0.001, $\eta_{p}^{2}$ = 0.334), where sham pMTG led to smaller P2 responses than bilateral pMTG (*p* < 0.001, Cohen's *d* = 0.718), left pMTG (*p* < 0.001, Cohen's *d* = 0.550), and right pMTG (*p* = 0.003, Cohen's *d* = 0.776). There was also a significant main effect of electrode site (*F*(2, 42) = 6.176, *p* = 0.013, $\eta_{p}^{2}$ = 0.227), showing larger P2 amplitudes at the fronto‐central electrodes compared to the frontal (*p* = 0.033, Cohen's *d* = 0.190) and central electrodes (*p* < 0.001, Cohen's d = 0.220). However, there was no significant main effect of perturbation direction (*F*(1, 21) = 0.321, *p* = 0.577, $\eta_{p}^{2}$ = 0.015) as well as significant interactions between any of three factors (*p*s > 0.05, all $\eta_{p}^{2}$ < 0.172).

Regarding the P2 latencies, there were no significant main effects of stimulation session (*F*(3, 63) = 1.670, *p* = 0.182, $\eta_{p}^{2}$ = 0.074), electrode site (*F*(2, 42) = 1.304, *p* = 0.274, $\eta_{p}^{2}$ = 0.058), or perturbation direction (*F*(1, 21) = 3.178, *p* = 0.089, $\eta_{p}^{2}$ = 0.131). In addition, there were no significant interactions between any of three factors (*p*s > 0.05, all $\eta_{p}^{2}$ < 0.137).

### 3.3 Source Localization

**FIGURE 4:**
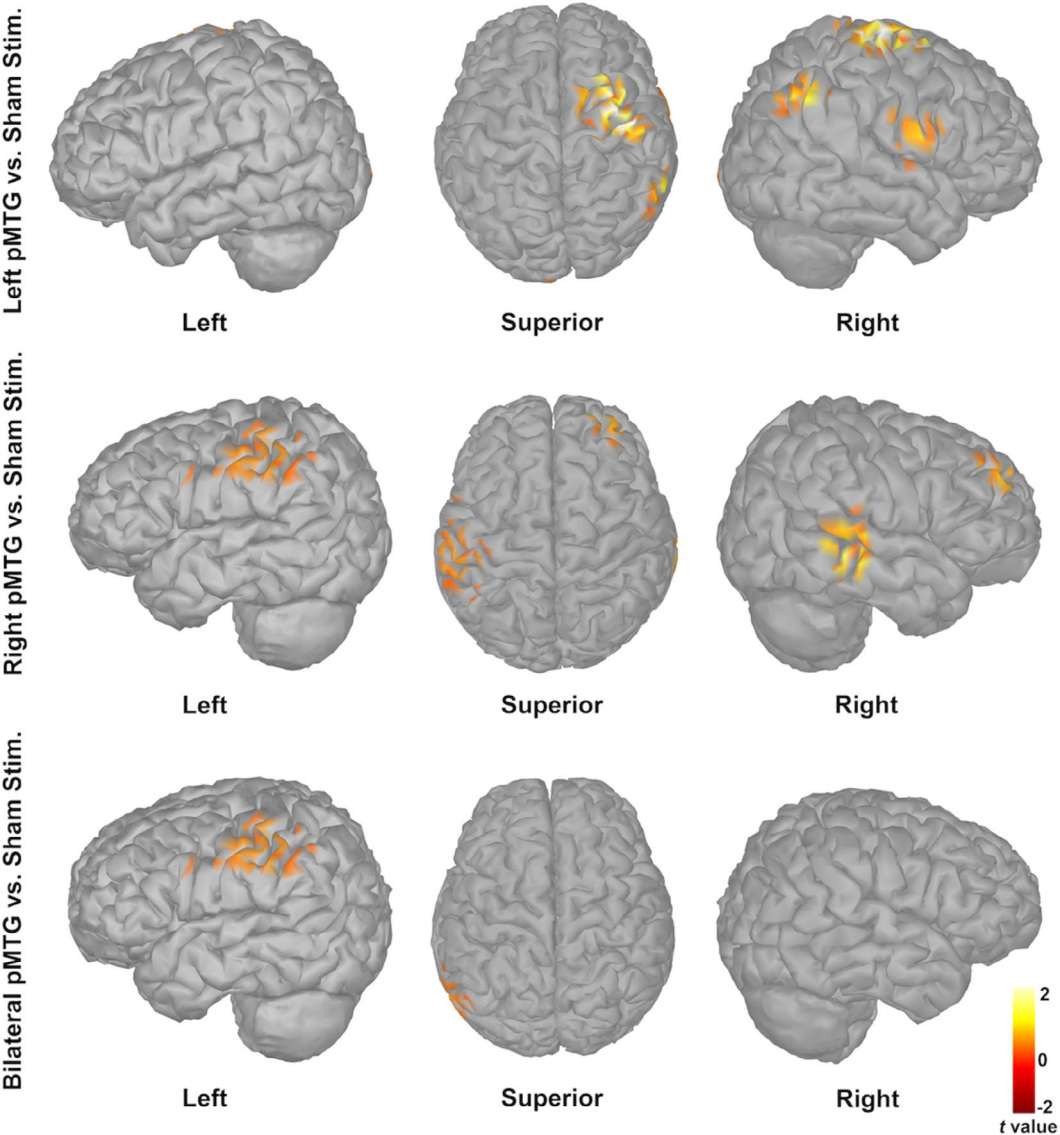
Grand‐averaged brain maps representing significant differences in cortical responses to pitch perturbations within the P2 time window between c‐TBS over the left (top), right (middle), or bilateral pMTG (bottom) relative to sham stimulation. Results are projected onto the left, superior, and right three‐dimensional views of a standard anatomical template. Positive *t*‐values indicate greater cortical activity under active stimulation relative to sham stimulation.

Source localization analyses revealed distinct cortical networks underlying enhanced P2 responses following c‐TBS over the left, right, and bilateral pMTGs compared to sham stimulation (Figure 4 and Table 1). Specifically, enhanced P2 responses following c‐TBS over the left pMTG were source‐localized in the right PMC (Brodmann area [BA] 4/6, *p* < 0.05), superior parietal lobule (SPL; BA 5/7, *p* < 0.001), and IPL (BA 39/40, *p* < 0.001). In contrast, enhanced P2 responses following c‐TBS over the right pMTG received significant contributions from the left PMC (BA 4/6, *p* < 0.05), right medial prefrontal cortex (mPFC; BA 9/10/11, *p* < 0.05), right STG (BA 22/41/42, *p* < 0.001), and right MTG (BA 21/37, *p* < 0.001). In addition, enhanced P2 responses following c‐TBS over bilateral pMTGs engaged areas primarily within the left IPL (BA 39/40, *p* < 0.05).

**TABLE 1:** t statistics for maximum activations obtained from comparisons between c‐TBS over the left, right, or bilateral pMTG and sham stimulation at P2 latency (MNI coordinates); threshold for significance at corrected *p* < 0.05.

Condition	BA	Brain region	*t*‐value	*X*	*Y*	*Z*	*p*
Left vs. Sham pMTG	5/7	Right SPL	1.92	−20	−24	75	< 0.001
	39/40	Right IPL	1.81	−55	−62	40	< 0.001
	4/6	Right PMC	1.63	−64	−2	30	< 0.05
Right vs. Sham pMTG	9/10/11	Right mPFC	1.62	−32	56	26	< 0.05
	22/41/42	Right STG	1.81	−69	−42	14	< 0.001
	21/37	Right MTG	1.85	−72	−34	−8	< 0.001
	4/6	Left PMC	1.57	58	−16	50	< 0.05
Bilateral vs. Sham pMTG	39/40	Left IPL	1.62	58	−50	45	< 0.05
	39	Left IPL	1.62	55	−64	32	< 0.05

Abbreviations: IPL, inferior parietal lobule; mPFC, medial prefrontal cortex; PMC, premotor cortex; pMTG, posterior middle temporal gyrus; SPL, superior parietal lobule; STG, superior temporal gyrus.

## 4 Discussion

The present study employed dual‐site c‐TBS over unilateral and bilateral pMTGs to investigate their causal contributions and interhemispheric dynamics in auditory‐motor control of vocal production. The results showed that c‐TBS over the left, right, or bilateral pMTG led to reduced peak magnitudes and shortened peak times of vocal compensations for pitch perturbations, accompanied by enhanced cortical P2 responses, compared to sham stimulation. These findings suggest that either the left or right pMTG is sufficient to support later‐stage auditory‐vocal integration. Reduced N1 responses were also observed only when c‐TBS was applied over the bilateral pMTG, suggesting interhemispheric coordination during early error monitoring. While neurobehavioral results did not differ significantly across all active stimulation conditions, source localization analyses revealed distinct neural networks, including the PMC, SPL, IPL, mPFC, MTG, and STG, underlying P2 enhancements induced by stimulation over different sites. By directly modulating bilateral pMTG activity, these findings provide novel causal evidence that the left and right pMTGs contribute to auditory‐motor integration for vocal feedback control through temporally specific interhemispheric interactions.

Behaviorally, we found reduced magnitudes and shortened latencies of vocal responses to pitch perturbations following c‐TBS over the left, right, or bilateral pMTG. Similar patterns have been reported in studies that applied unilateral c‐TBS over the left SMA (Dai, Chen, et al. 2022; Dai, Wang, et al. 2022), right STG (Liu et al. 2023), and left/right SMG (Li, Chang, et al. 2023; Li, Zhu, et al. 2023), whereas opposite effects were observed following c‐TBS over the left DLPFC (Liu et al. 2020). These findings highlight the functional specialization of cortical regions in vocal feedback control. One interpretation of smaller and/or faster vocal responses is a shift toward greater reliance on feedforward control, thereby reducing dependence on feedback control (Golfinopoulos et al. 2010; Houde et al. 2019; Scheerer and Jones 2012, 2014). This perspective is supported by evidence from clinical populations, where enhanced and/or prolonged vocal compensations are commonly observed in individuals with impaired sensorimotor integration due to Parkinson's disease (PD) (Huang et al. 2016; Liu et al. 2012; Mollaei et al. 2016), AD (Ranasinghe et al. 2017), spinocerebellar ataxia (SCA) (Houde et al. 2019; Li et al. 2019), and stroke (Johnson et al. 2020), reflecting an overreliance on auditory feedback in the presence of impaired feedforward mechanisms. Importantly, normalization of exaggerated vocal compensations has been observed in individuals with PD following extensive voice treatment (Li et al. 2021), c‐TBS over the left SMA (Dai, Chen, et al. 2022; Dai, Wang, et al. 2022), and deep brain stimulation (Behroozmand et al. 2019), as well as in individuals with SCA following c‐TBS over the right cerebellum (Lin et al. 2022). Within this framework, c‐TBS over the left, right, or bilateral pMTG may similarly reweight the balance between feedforward and feedback control in auditory‐vocal integration, reducing reliance on feedback control and thereby decreasing vocal compensations. Given that lesion evidence has revealed a link between stroke‐induced damage to the left MTG and STG and attenuated vocal compensations for pitch perturbations within the onset window of 50–150 ms (Behroozmand et al. 2018), however, we cannot rule out the possibility that c‐TBS applied to the pMTG may disrupt the neural processes required for online detection and/or correction of auditory feedback errors. Such disruption may result in a diminished capacity to engage feedback‐based mechanisms for fine‐tuning vocal adjustment.

Systematic changes in the cortical N1 and P2 responses were also observed when dual‐site c‐TBS was applied over the pMTG. Compared to sham stimulation, c‐TBS over the bilateral pMTG significantly decreased N1 responses. This observation parallels previous findings showing reduced N1 responses following c‐TBS over the left SMA (Dai, Chen, et al. 2022; Dai, Wang, et al. 2022), right STG (Liu et al. 2023), and right cerebellum (Lin et al. 2022). The N1 response is typically regarded as an index of early error detection in vocal feedback control (Behroozmand et al. 2014; Korzyukov et al. 2012), with primary neural generators localized in the MTG and STG (Guo et al. 2017). Consistent with this role, larger pitch perturbations consistently elicit greater N1 responses (Behroozmand et al. 2009; Liu et al. 2011; Scheerer et al. 2013). One interpretation of reduced N1 responses is that they may reflect enhanced neural efficiency in processing auditory feedback. Empirical support for this account comes from studies showing reduced N1 responses during vocal pitch regulation in participants undergoing auditory training and speech‐sound learning (Chen et al. 2015; Li, Guo, et al. 2015; Li, Qu, et al. 2015). These reductions are thought to arise from more precise encoding of auditory feedback errors with fewer neural resources, representing a reorganization toward more efficient auditory processing (Gervain and Geffen 2019; Zatorre et al. 2012). In the present study, bilateral pMTG stimulation may induce a functional redistribution across hemispheres, facilitating interhemispheric coordination that supports accurate error detection. Such a reconfiguration may reduce redundancy in auditory inputs and promote more efficient encoding of vocal feedback errors. This interpretation aligns with the critical role of the pMTG in the temporal processing of vocal output errors (Behroozmand et al. 2018; Guo et al. 2017; Kort et al. 2016). Alternatively, the observed N1 reduction may reflect a disruption of error detection processes during vocal feedback control, as c‐TBS is typically known to exert inhibitory effects on cortical excitability. This interpretation is in line with lesion evidence that structural damage to the left MTG and STG leads to reduced N1 responses to pitch perturbations (Behroozmand et al. 2022), suggesting that bilateral temporal lobe integrity is essential for early‐stage error monitoring.

In addition, c‐TBS over either the left or right pMTG did not produce significant changes in N1 responses, suggesting that early error detection in vocal feedback control may be resilient to disruption in a single hemisphere. This resilience may reflect interhemispheric coordination, such as functional compensation, wherein the unaffected hemisphere can upregulate its activity or reorganize functional networks to preserve task performance when its counterpart is perturbed or damaged (Grefkes and Fink 2011; Hartwigsen et al. 2013). In this context, inhibitory c‐TBS over one pMTG may be counteracted by compensatory activity in the contralateral pMTG, maintaining the functional integrity of the feedback error detection system. The bilateral organization of the auditory system further supports this resilience, as both hemispheres are capable of processing auditory feedback (Hickok and Poeppel 2007; Zatorre et al. 2002), allowing the unaffected hemisphere to compensate for disruptions in the stimulated hemisphere. Therefore, c‐TBS over the left or right pMTG may not be sufficient to disrupt interhemispheric coordination to a degree that induces changes in the N1 responses, further supporting the functional dynamics of the bilateral pMTG in early error detection during vocal feedback control.

The P2 response has been considered as a neural correlate of sensorimotor integration during speech production, reflecting cortical mechanisms that support continuous monitoring and rapid correction of voice feedback errors (Behroozmand et al. 2014, 2011; Guo et al. 2017; Liu et al. 2023, 2020). In the present study, c‐TBS over the left, right, or bilateral pMTG led to significantly increased P2 responses compared to sham stimulation, suggesting the involvement of both the left and right pMTGs in later‐stage processing of vocal feedback errors. In line with our finding, Kort et al. (2016) identified peak activation in the left and right MTGs in response to pitch perturbations within the 175–225 ms time window, a period closely associated with the P2 response. Similarly, Ranasinghe et al. (2019) observed increased cortical activity in the right posterior middle temporal cortex within the 200–300 ms time window, which was significantly correlated with peak magnitude of vocal response to pitch perturbations. Converging evidence from source localization has also identified the left and right MTGs as primary generators of P2 response during auditory feedback processing (Dai, Chen, et al. 2022; Dai, Wang, et al. 2022; Guo et al. 2017; Huang et al. 2016). Taken together with these findings, our results provide causal evidence for the essential role of the left and right pMTGs in auditory–motor integration for correcting voice feedback errors.

Although significant differences in P2 responses were not found across the three stimulation conditions, this pattern does not imply functional independence between the left and right pMTGs. Rather, it is more consistent with the notion of functional redundancy, wherein either hemisphere is sufficient in supporting later‐stage auditory‐motor integration. Such redundancy does not preclude interhemispheric coordination but reflects a dynamic reorganization of neural resources to support vocal feedback control under unilateral or bilateral pMTG stimulation. This interpretation is substantiated by source localization results, which revealed distinct neural networks underlying P2 enhancements for each stimulation condition. Specifically, following c‐TBS over the left pMTG, P2 enhancement was associated with increased contralateral activity in the right PMC, SPL, and IPL, suggesting compensatory recruitment of right motor‐parietal regions to facilitate sensorimotor integration for error correction (Tourville and Guenther 2011). In contrast, c‐TBS over the right pMTG resulted in P2 enhancement linked to increased activity in the left PMC and ipsilateral right mPFC, STG, and MTG, suggesting ipsilateral auditory processing and left‐hemisphere motor control (Zarate 2013). Notably, P2 enhancement following c‐TBS over the bilateral pMTG predominantly engaged the left IPL, a region that is essentially involved in auditory‐motor transformation during speech production (Hickok et al. 2003; Li, Chang, et al. 2023; Li, Zhu, et al. 2023). This selective engagement of the left IPL may reflect a compensatory reorganization of the speech monitoring network, whereby parietal regions play a more central role in integrating auditory feedback with motor commands when the bilateral pMTG is disrupted. These distinct activation patterns reflect the dynamic interplay between the left and right pMTGs, highlighting their roles as integral components of a distributed neural network that modulates vocal feedback control through interhemispheric coordination and functional specialization.

Interestingly, c‐TBS over the left, right, or bilateral pMTG led to increased, rather than reduced, P2 responses to vocal pitch perturbations. A similar pattern was also found following c‐TBS over the right pSTG (Liu et al. 2023), emphasizing the essential role of the auditory cortex in vocal feedback control (Chang et al. 2013; Eliades and Wang 2008; Kort et al. 2016; Parkinson et al. 2012). Concurrent TMS‐fMRI studies have shown that c‐TBS over a targeted region can lead to increased activity in the contralateral hemisphere as a compensatory response to TMS‐induced disruption (Andoh and Paus 2011; Andoh and Zatorre 2013; Hartwigsen et al. 2013). Notably, increased P2 responses following c‐TBS over the right STG were primarily driven by ipsilateral rather than contralateral regions (Liu et al. 2023), supporting a right‐lateralized network in coordinating motor adjustments to vocal feedback errors (Kort et al. 2016; Tourville et al. 2008). In the present study, increased P2 responses following pMTG stimulation were source‐localized in the motor, parietal, and prefrontal regions, suggesting compensatory recruitment of ipsilateral or contralateral regions in later‐stage auditory‐motor integration. This pattern is consistent with the notion that TMS‐induced disruptions can facilitate functional reorganization and enhance cortico‐cortical connectivity (Siebner et al. 2009).

Similar P2 enhancements have also been observed following perceptual learning, music training, and voice treatment (Behroozmand et al. 2014; Guo et al. 2017; Li, Guo, et al. 2015; Li, Qu, et al. 2015; Li et al. 2021). Moreover, significant negative correlations have been found between P2 amplitude and vocal compensation magnitude (Huang et al. 2019; Lin et al. 2022; Liu et al. 2023; Scheerer et al. 2013). These findings indicate that greater cortical activity in the P2 time window is associated with smaller vocal compensations for feedback perturbation. This relationship suggests that increased P2 response may reflect more efficient neural encoding of auditory feedback and motor adjustment, enabling rapid error detection and correction while preventing excessive compensatory behavior (Behroozmand et al. 2014; Guo et al. 2017; Liu et al. 2023; Zarate and Zatorre 2008). In this context, our observation of P2 enhancement following pMTG stimulation may represent a reorganization of the audio‐vocal system, in which discrepancies between intended and actual vocal output are encoded with greater neural salience to facilitate fine‐tuned motor adjustment. Such reorganization may be driven by a recalibration of the feedback‐feedforward balance, reducing reliance on feedback mechanisms while strengthening dependence on feedforward mechanisms. This shift may serve as a compensatory strategy to maintain speech monitoring and control under transient disruption of temporal lobe function.

It is noteworthy that both theoretical and empirical findings have emphasized a right‐lateralized network, particularly involving the ventral PMC, posterior STG, and IFG, that plays a central role in auditory feedback control of vocal production (Golfinopoulos et al. 2010; Houde and Chang 2015; Liu et al. 2023; Tourville and Guenther 2011; Tourville et al. 2008). However, accumulating evidence has shown a more distributed architecture, where the left‐lateralized or bilateral regions, such as the DLPFC, IFG, PMC, SMA, STG, and IPL/SMG, also contribute significantly to generating vocal compensations for perturbed auditory feedback (Behroozmand et al. 2020, 2018; Dai, Chen, et al. 2022; Dai, Wang, et al. 2022; Kort et al. 2014; Li, Chang, et al. 2023; Li, Zhu, et al. 2023; Scott et al. 2020). Our work extends these findings by providing causal evidence for bilateral pMTG involvement in vocal feedback control, advancing the bihemispheric dynamics of auditory‐vocal integration. More importantly, our results reveal the temporal dynamics of interhemispheric interaction in the bilateral pMTG involvement. Specifically, reduced N1 responses were observed only following c‐TBS over the bilateral pMTG, suggesting early‐phase error detection requires interhemispheric coordination between the left and right pMTGs. In contrast, enhanced P2 responses were found across unilateral and bilateral stimulation conditions, suggesting that either the left or right pMTG may be sufficient to sustain later‐phase auditory‐motor integration. This dissociation suggests a temporal shift in interhemispheric interaction between the pMTGs, from bilateral coordination during early error detection to unilateral sufficiency during subsequent error correction. This pattern is in line with previous research on vocal pitch regulation by Kort et al. (2016), who reported that the left MTG was predominantly activated during early sensory detection while bilateral MTG engagement became prominent during later motor coordination. Together, our results refine current models of speech motor control by showing that the pMTG is not only bilaterally engaged but also dynamically coordinated in a phase‐specific manner, providing novel insights into the interhemispheric dynamics underlying auditory‐vocal integration.

Several limitations of the present study should be acknowledged. First, although c‐TBS over the bilateral pMTG elicited significant reductions in N1 responses, source localization did not reveal their neural generators. This may be due to the relatively small amplitude and modest effect size of the N1 component that limits the sensitivity of source reconstruction, particularly under conservative corrections for multiple comparisons. Second, while our findings suggest possible interhemispheric coordination during vocal feedback control, we did not obtain direct evidence of functional compensation such as ipsilateral suppression or contralateral enhancement. Future studies combining TMS with high‐resolution neuroimaging techniques, such as fMRI or MEG, will be needed to determine whether inhibitory or compensatory mechanisms operate between the left and right pMTG during vocal feedback control.

## 5 Conclusions

By employing dual‐site c‐TBS over the left, right, and bilateral pMTG, the present study found reduced magnitudes and shortened latencies of vocal responses, accompanied by enhanced P2 responses originating from distinct neural networks in the presence of pitch perturbations. Reduced N1 responses were also observed only following c‐TBS over the bilateral pMTG. These findings provide direct evidence supporting the causal roles of both the left and right pMTGs in vocal motor control mediated by auditory feedback. Importantly, our results are indicative of a phase‐specific interhemispheric interaction between the left and right pMTGs, characterized by coordinated activity during early error monitoring and independent processing during later auditory‐motor integration, advancing our understanding of the functional specialization and interaction of the bilateral pMTG in the bihemispheric dynamics of vocal feedback control.

## Author Contributions

Q.L. conceptualized the study, acquired data, conducted formal analysis, and wrote the original draft. J.L. contributed to data curation, acquisition, and formal analysis. S.Z. participated in data acquisition and formal analysis. M.C. and X.H. assisted with data acquisition. D.L., J.L., X.W., Y.L., and X.C. secured funding. P.L., G.D., and H.L. contributed to conceptualization, funding acquisition, critical revision of the manuscript, and provided final approval of the submitted version. All authors reviewed and approved the manuscript.

## Conflicts of Interest

The authors declare no conflicts of interest.

## Data Availability

The data that support the findings of this study are available on request from the corresponding author. The data are not publicly available due to privacy or ethical restrictions.
